# Comparative Immunohistochemical Analysis of FAP and c-MET Expression Across Renal Cell Carcinoma Subtypes: Preliminary Results and Potential Theranostic Implications

**DOI:** 10.3390/diagnostics16193228

**Published:** 2026-10-06

**Authors:** Lena M. Unterrainer, Adrien Holzgreve, Sophie C. Siegmund, Magdalena Schöll, Julia Schwenke, Rudolf A. Werner, Frederick Klauschen, Michael Staehler, Stephan T. Ledderose

**Affiliations:** 1 Department of Nuclear Medicine, LMU University Hospital, LMU Munich, 91377 Munich, Germany; 2 Institute of Pathology, Faculty of Medicine, LMU Munich, 80337 Munich, Germany; 3 Department of Urology, LMU University Hospital, LMU Munich, 81377 Munich, Germany

**Keywords:** cMET, FAP, theranostics, RCC, immunohistochemistry

## Abstract

**Background**: Renal cell carcinoma (RCC) comprises multiple histopathological subtypes with distinct biological characteristics and clinical behavior. Despite advances in conventional imaging, the detection of small RCC lesions or distant metastases remains challenging and the prognosis of advanced RCC is still poor. Theranostic approaches are promising tools combining diagnostic imaging and targeted radionuclide-based cancer therapy. The identification of suitable molecular targets in tumor tissue is a major focus of current research. Fibroblast activation protein (FAP) and hepatocyte growth factor receptor (c-MET) have been proposed as theranostic targets in several solid cancers, including RCC. However, available data are limited and inconsistent, particularly regarding expression patterns across different RCC subtypes. **Methods**: In this study, we evaluated the expression patterns of FAP and c-MET immunohistochemically across major RCC subtypes (clear cell RCC [ccRCC], papillary RCC [pRCC], and chromophobe RCC [cpRCC]). Staining intensity was assessed semi-quantitatively and correlated with histological RCC subtypes. **Results**: FAP expression differed substantially among RCC subtypes, being highest in ccRCC, significantly lower in pRCC, and largely absent in cpRCC. In contrast, c-MET was ubiquitously expressed across all three RCC subtypes but showed a lower specificity for tumor cells. **Conclusions**: Our results provide a rationale for further evaluating FAP and c-MET as potential theranostic targets across RCC subtypes and establishing a foundation for future RCC-subtype-specific imaging-pathology correlation studies. Validation in larger multicenter cohorts including paired molecular imaging data is required before their clinical translation can be considered.

## 1. Introduction

Renal cell carcinoma (RCC) is the most common malignant tumor of the kidney, accounting for approximately 4% of all adult cancers [1]. The most prevalent histological subtype is clear cell RCC (ccRCC), followed by papillary RCC (pRCC) and chromophobe RCC (cpRCC), each characterized by distinct genetic, morphological and clinical features [2,3]. While surgical resection is the standard treatment in localized disease, a substantial proportion of patients suffer from recurrent cancer or progress to advanced disease [4]. For proper clinical management, it is essential to detect recurrent or metastatic RCC as early as possible [5,6]. Current guidelines recommend the use of contrast-enhanced computed tomography (CT) or magnetic resonance imaging (MRI), but overall sensitivity of these conventional imaging methods is limited [5]. Sensitivity is usually higher with ^18^F-fluoro-2-deoxy-2-D-glucose (18F-FDG) positron emission tomography (PET). However, this plays only a limited role in RCC, where the renal excretion of the radiotracer hampers accurate scan interpretation [7]. Given the poor prognosis of advanced RCC, there is a need for improved diagnostic and therapeutic strategies [4].

In recent years, theranostics, a strategy combining molecular imaging and targeted therapy using the same radioligand, has gained increasing relevance in oncology [8]. The identification of suitable targets that exhibit differential expression between tumor and normal tissues is a critical step in the development of successful theranostic approaches [9]. For example, the use of prostate specific membrane antigen (PSMA) is well-established for theranostic approaches in prostate cancer [10]. Recently, PSMA-directed radioligands were demonstrated to also be of potential value for the diagnosis and therapy of RCC [11,12,13,14,15]. However, given the intrinsic biological heterogeneity of RCC subtypes, an accurate subtype-specific characterization of potential target molecules at the tissue level is essential for further translational theranostic research on these cancer entities [6].

Fibroblast activation protein (FAP) is a membrane-bound serine protease that is mainly expressed by cancer-associated fibroblasts and largely absent from most normal tissues [16]. Its predominant localization within the tumor stroma and its association with tissue remodeling, tumor progression and invasion make FAP an attractive target for both molecular imaging and radionuclide therapy [16,17,18,19,20]. Preliminary studies suggest that ^68^Ga-FAP inhibitor (FAPI) PET/CT may improve visualization of small RCC lesions or metastatic deposits [21,22,23]. However, FAPI uptake varies considerably in RCC cohorts, and data on FAP expression across different RCC subtypes remains limited and inconsistent [21,24,25,26,27,28].

The hepatocyte growth factor receptor c-MET is a transmembrane tyrosine kinase, which plays a central role in organ homeostasis and tissue remodeling and is physiologically expressed in multiple tissues, including the kidney [29,30]. Aberrant activation of the c-MET signaling pathway has been implicated in the pathogenesis of RCC and is associated with tumor progression and resistance to therapy [31]. As c-MET is directly expressed by malignant tumor cells, it represents an established therapeutic target of tyrosine kinase inhibitors like cabozantinib [32,33]. In addition, c-MET may function as a potential theranostic target [34].

FAP and c-MET address different spatial compartments of RCC: the tumor-adjacent stroma as part of the tumor microenvironment (FAP) and the tumor cells themselves (c-MET). A systematic immunohistochemical characterization of their expression patterns across RCC subtypes is important to determine whether FAP- and cMET-directed theranostic approaches are likely to have broad applicability across RCCs or whether they might be particularly relevant to specific subtypes. However, current data on expression profiles of FAP and c-MET in different RCC subtypes is limited [24,25,26,27,28,35]. In this study, we evaluated FAP and c-MET protein expression immunohistochemically in ccRCC, pRCC and cpRCC. We found distinct differences in FAP and c-MET expression patterns across RCC subtypes. High expression levels in ccRCC and pRCC suggest that FAP might be particularly useful as a theranostic target in these RCCs, while c-MET showed broad expression across all RCC subtypes but lower specificity for tumor cells.

## 2. Materials and Methods

The study was approved by the medical ethics committee of LMU Munich (# 21-0310, date of approval: 19 April 2021) and carried out in compliance with the principles outlined in the Declaration of Helsinki. The requirement of informed consent was waived in accordance with the institutional guidelines for retrospective studies using fully anonymized left-over diagnostic tissue samples and associated clinicopathological data stripped of all direct identifiers.

### 2.1. Tissue Samples

This retrospective study was conducted using tissue samples of 65 patients who underwent surgery for RCC at the Department of Urology, LMU Munich, between 2011 and 2019, as previously described [6]. Briefly, formalin-fixed and paraffin-embedded (FFPE) samples from three predefined RCC subtypes (ccRCC, pRCC and cpRCC) were selected using a stratified sampling approach to ensure comparability among these subgroups. Because pRCC is no longer subdivided into type 1 and type 2 according to the current WHO classification, only pRCCs with classic histomorphology (previously type 1) were included in this study (former type 2 pRCC are now viewed as different entities, such as fumarate hydratase-deficient RCC) [36]. Exclusion criteria were an unclear histological diagnosis preventing a definitive subtype classification, insufficient tumor tissue for the planned immunohistochemical analyses, and missing information regarding TNM stage. All available tumor sections were examined using haematoxylin and eosin (H&E)-stained slides by board-certified pathologists at the Institute of Pathology (LMU Munich). Tumors were (re-)classified according to the current WHO Classification of Tumors, Fifth Edition, and ccRCCs and pRCCs were graded according to the standardized WHO Classification of Tumors/International Society of Urological Pathology (WHO/ISUP) histological grading system of renal cell tumor types (for cpRCC, there is no established WHO/ISUP grading system to date) [36,37]. A representative section was chosen for each case, ensuring the presence of both tumor tissue and normal kidney tissue for further analysis.

### 2.2. Immunohistochemical Analysis

Immunoreactivity for FAP and cMET in ccRCC, pRCC and cpRCC was examined immunohistochemically on FFPE tumor tissue samples. Antigen retrieval was conducted via heat treatment using Target Unmasking Fluid (PanPath B.V., Budel, The Netherlands; Z000R0000) for FAP and Antigen Retrieval AR-10 (DCS Diagnostics, Hamburg, Germany; HK057-5KE) for c-MET staining. Slides were subsequently incubated with rabbit monoclonal anti-human primary antibodies against FAP (1:120; Abcam, Berlin, Germany; EPR20021) or c-MET (1:250; Abcam, Berlin, Germany; EP1454Y) at room temperature for 60 min. Bound antibodies against FAP were detected with ImmPRESS Anti-Rabbit IgG Polymer Kit (Vector Laboratories, Newark, CA, USA; MP-7401). The Vectastain ABC-Kit Elite Universal (Vector Laboratories; PK6200) in combination with a streptavidin/biotin blocking kit (Vector Laboratories; SP-2002) was used as to detect c-MET staining. Staining results were visualized using DAB+ (Agilent Technologies; Santa Clara, CA, USA; K3468) as a chromogen. Sections were finally counterstained with hematoxylin (Vector Laboratories; H-3401), dehydrated, and mounted.

### 2.3. Semi-Quantitative Analysis of FAP and c-MET Expression

**Figure 1:**
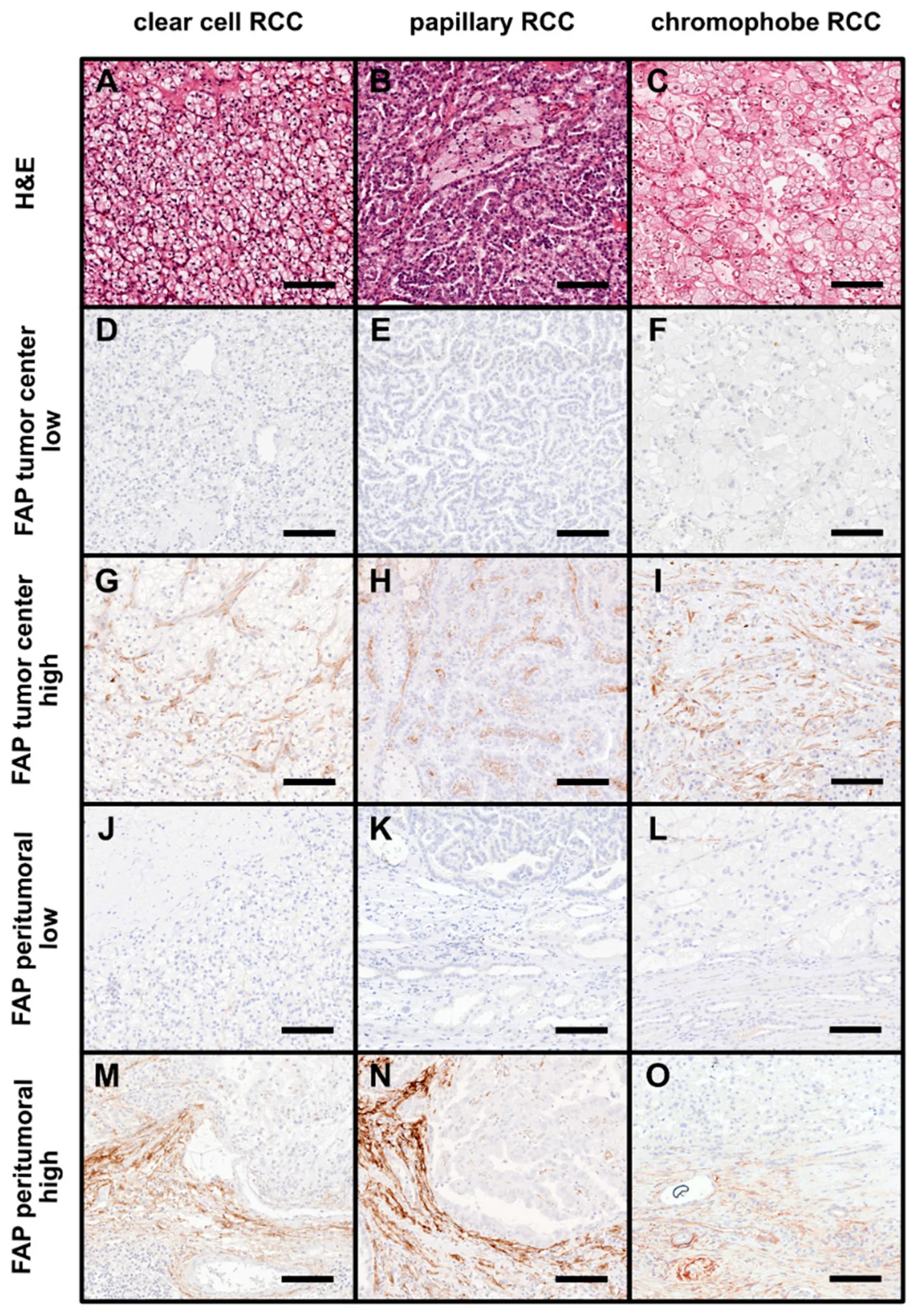
FAP expression across histopathological subtypes of renal cell carcinoma (RCC). (**A**–**C**) Representative hematoxylin and eosin (H&E)–stained micrographs illustrating the characteristic histomorphological features of clear cell RCC (ccRCC, (**A**)), papillary RCC (pRCC, (**B**)), and chromophobe RCC (cpRCC, (**C**)). (**D**–**I**) Representative images of FAP-immunostained sections of ccRCC (**D**,**G**), pRCC (**E**,**H**), and cpRCC (**F**,**I**) showing low (**D**–**F**) and high (**G**–**I**) levels of FAP immunoreactivity within the tumor center. (**J**–**L**) Representative examples of low FAP expression in the peritumoral region of ccRCC (**J**), pRCC (**K**), and cpRCC (**L**). (**M**–**O**) Representative micrographs demonstrating high peritumoral FAP expression in ccRCC (**M**), pRCC (**N**), and cpRCC (**O**). All images were acquired at 20× magnification; scale bar = 100 µm.

FAP staining was assessed as immunoreactivity score (IRS) as previously described [38,39]. Briefly, the percentage of immunoreactive stromal cells was scored semi-quantitatively as 0 (absent or <1%), 1 (1–25%), 2 (36–50%), 3 (51–75%), or 4 (76–100%). The staining intensity was also graded semi-quantitatively on a scale reaching from 0 to 3 (0 = absent staining, 1 = weak staining, 2 = moderate staining, 3 = strong staining). The IRS was then calculated as the product of both values. To capture the heterogeneity of protein expression throughout the entire tumor, the IRS was determined both in the tumor center and at the invasive front [40]. The invasive front was defined as the tumor area within one high-power field (HPF, 40× lens) that directly bordered normal renal parenchyma. To account for both tumor compartments while maintaining the original range of the IRS scale, we subsequently calculated an overall IRS as the arithmetic mean of the tumor center and invasive front IRS values [41]. Since PET/CT imaging has a considerably lower spatial resolution than immunohistochemistry, this overall IRS is assumed to most closely reflect the expected FAP uptake in PET imaging and was used for statistical analyses [42]. Representative examples of different FAP staining patterns are shown in Figure 1.

**Figure 2:**
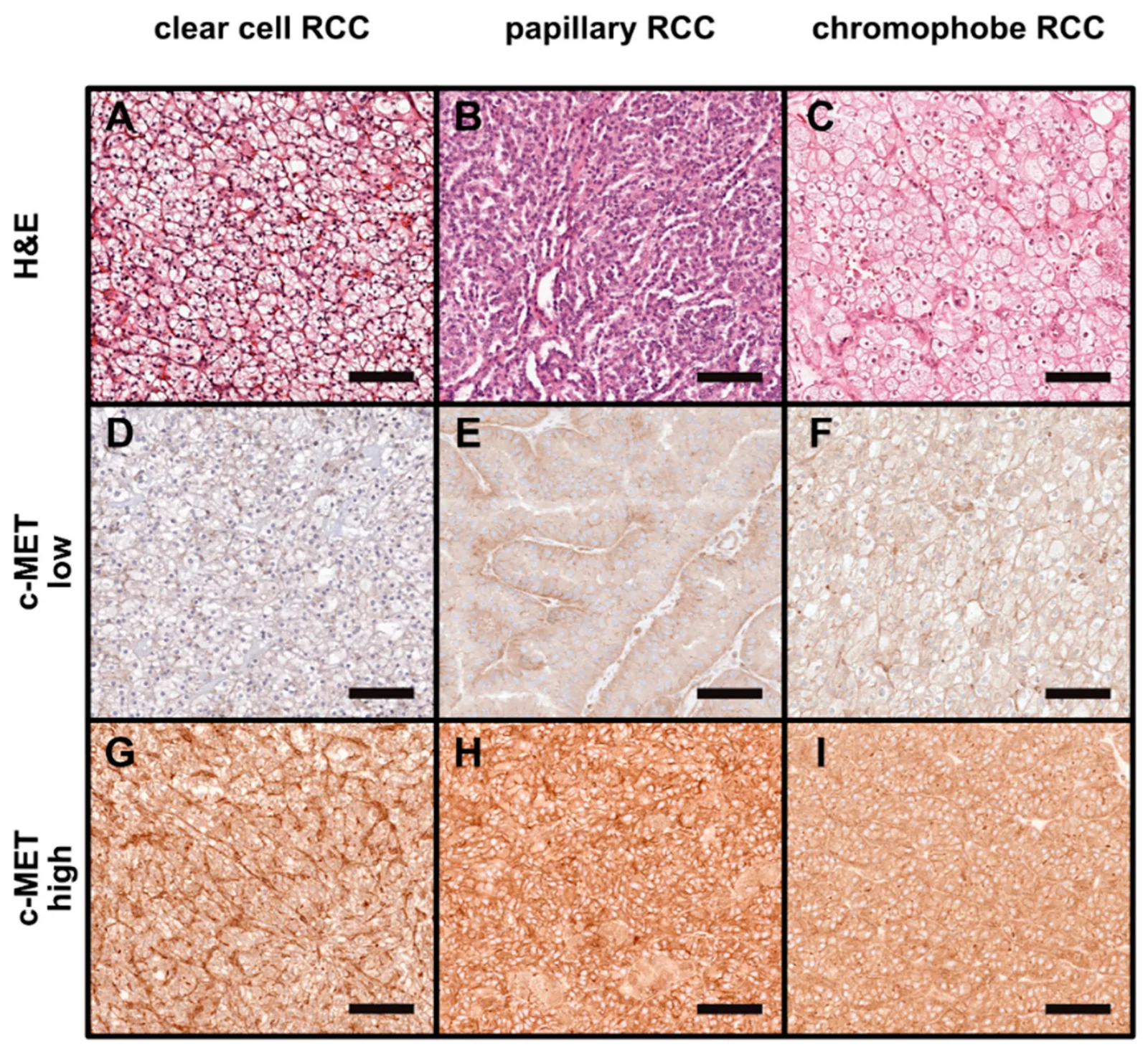
c-MET expression across histopathological subtypes of renal cell carcinoma (RCC). (**A**–**C**) Representative hematoxylin and eosin (H&E)–stained micrographs showing the typical histomorphological features of clear cell RCC (ccRCC, (**A**)), papillary RCC (pRCC, (**B**)), and chromophobe RCC (cpRCC, (**C**)). (**D**–**F**) Representative images of c-MET–immunostained sections of ccRCC (**D**), pRCC (**E**), and cpRCC (**F**) showing low c-MET immunoreactivity. (**G**–**I**) Representative examples demonstrating high c-MET expression in ccRCC (**G**), pRCC (**H**), and cpRCC (**I**). All images were acquired at 20× magnification; scale bar = 100 µm.

To ensure comparability with previous studies, c-MET staining was evaluated using the histochemical scoring system (H-score) [43]. Briefly, this scoring system incorporates staining intensity as well as the proportion of stained cells at each intensity level [44]. Staining intensity is scored as 0 (no evidence of staining), 1 (weak staining), 2 (moderate staining) and 3 (strong staining). The H-score is finally calculated as the sum of all values and reaches from 0 to 300. Because the H-score is based on the evaluation of all tumor cells across the entire tumor, it inherently reflects an average expression level facilitating the comparison with imaging data [45]. Figure 2 shows representative examples of c-MET-stained tumor samples with different H-scores.

IRS and H-scores were assigned by a board-certified pathologist (SL) with extensive experience in the analysis and interpretation of immunohistochemical stains. The evaluation was blinded to patient data, clinical information, and TNM classification (blinding to the histological RCC subtype is not possible during immunohistochemical analysis). Difficult cases were jointly reviewed and discussed with another experienced pathologist (JS) to reach a consensus score.

### 2.4. Statistical Analyses

Statistical analyses were performed with GraphPad Prism (Version 9.5.0, GraphPad Software, La Jolla, CA, USA). To provide a descriptive overview of baseline patient characteristics according to c-MET and FAP expression, tumor samples were post hoc categorized into groups with low and high immunoreactivity based on cohort-specific median values of total IRS (FAP) and H-score (c-MET). Due to the small subgroup sizes and limited number of observations further statistical analyses of the clinicopathological characteristics stratified to FAP- or c-MET expression were not considered appropriate. Descriptive statistics are displayed as median and interquartile range (IQR). Differences in FAP and c-MET staining intensity between RCC subtype groups were compared using the Kruskal–Wallis test followed by post hoc Dunn’s test with Bonferroni adjustment for the three comparisons. All pairwise tests were two-sided and an adjusted *p*-value < 0.05 was considered statistically significant.

## 3. Results

### 3.1. Patient Characteristics

Patient characteristics have been published previously [6]. Briefly, the study included 65 patients who had undergone surgery for RCC with curative intent. Among the tumors, 21 were classified as ccRCC (32%), 24 as pRCC (37%), and 20 as cpRCC (30%). The average age of all patients at the time of surgery was 67 years (range: 36–90). The demographic and pathologic characteristics depending on FAP and c-MET expression are listed in Table 1 and Table 2.

### 3.2. FAP and c-MET Expression in Non-Neoplastic Kidney Tissue

**Figure 3:**
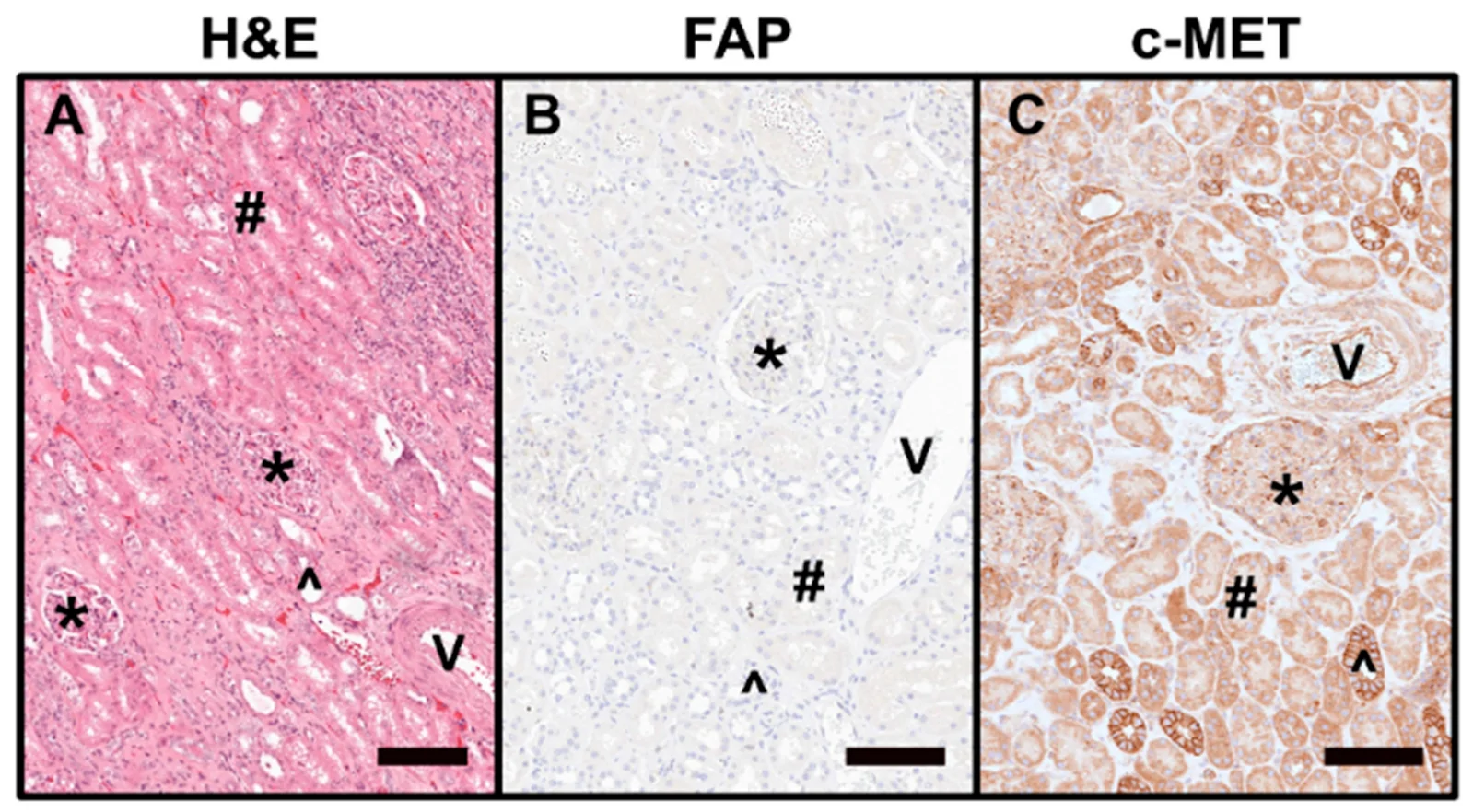
c-MET and FAP expression in non-neoplastic renal tissue. (**A**) Representative hematoxylin and eosin (H&E)–stained micrograph of normal renal parenchyma showing glomeruli (*), renal vessels (V), and proximal (#) and distal (^) tubules. (**B**) FAP expression is absent in non-neoplastic renal tissue. (**C**) Variable c-MET immunoreactivity is observed in epithelial cells of proximal and distal tubules, in the capillary loops of glomeruli, and in vascular structures. All images were acquired at 20× magnification; scale bar = 100 µm.

Tumor-free renal parenchyma was defined as micromorphologically regularly structured renal parenchyma with at least 2 mm distance from invasive cancer. c-MET showed membranous and cytoplasmic immunoreactivity in epithelial cells of proximal (moderate staining intensity) and distal tubule sections (strong staining intensity). In the capillary loops of the glomeruli, only a focal and weak immunoreactivity was observed. Endothelial cells in local arteries and veins as well as smooth muscle cells of the arterial walls also stained positive for c-MET. In contrast, there was no FAP expression in normal renal structures, including the fibrous renal capsule. Representative microphotographs of FAP and c-MET-stained non-neoplastic kidney tissues are depicted in Figure 3.

### 3.3. FAP Expression in Different Subtypes of RCC

In 17 of 21 (80.1%) examined ccRCC cases, FAP expression was observed in the endothelial cells of tumor-associated vessels and in cancer-associated fibroblasts outlining the tumor-cell lobes at the tumor center. The tumor cells themselves showed no staining for FAP. The intensity and quantity of stained cells varied between tumor samples, resulting in different IRS values (IRS tumor center: 4.0, IQR 2.0–6.0). At the invasive front, FAP expression was found in 19 (90.5%) ccRCC cases, mainly in the area of the fibrous tumor pseudocapsule (IRS peritumoral: 4.0, IQR, 2.0–6.0). A combined evaluation revealed a total IRS > 0 in 19 (90.5%) tumor samples (total IRS: 4.0, IQR 3.0–5.0), and 67% of the samples showed strong FAP expression with a total IRS ≥ 4.

In 10 out of 24 (41.7%) examined pRCC cases, FAP expression was observed at the tumor center (IRS tumor center: 0, IQR 0–1.3). Here, FAP was mainly found in stromal cells and vessels within the characteristic fibrovascular cores of this tumor entity. Similar to ccRCC, more tumor samples showed FAP expression at the invasion front (75%), with a higher IRS compared to the tumor center (IRS peritumoral: 1.5, IQR, 0.8–4.0). The combined analysis showed a total IRS > 0 in 20 tumor samples (83.3%; total IRS: 1.5, IQR, 0.9–2.0), and 54% of the samples revealed a comparatively strong FAP expression with a total IRS ≥ 1,5.

In cpRCC, FAP-positive stromal cells and vessels were found only focally in 2 out of 20 tumors at the tumor center (IRS tumor center: 0, IQR 0–0). In 5 tumor samples, FAP-positive stromal cells were found in focal areas peritumorally, but also with only weak staining intensity (IRS peritumoral: 0, IQR 0–0.3). Consequently, only 6 tumors showed a total IRS > 0 (IQR 0–0.5), and 70% of tumor samples were scored with a total IRS of 0, highlighting the low FAP expression in cpRCC.

**Figure 4:**
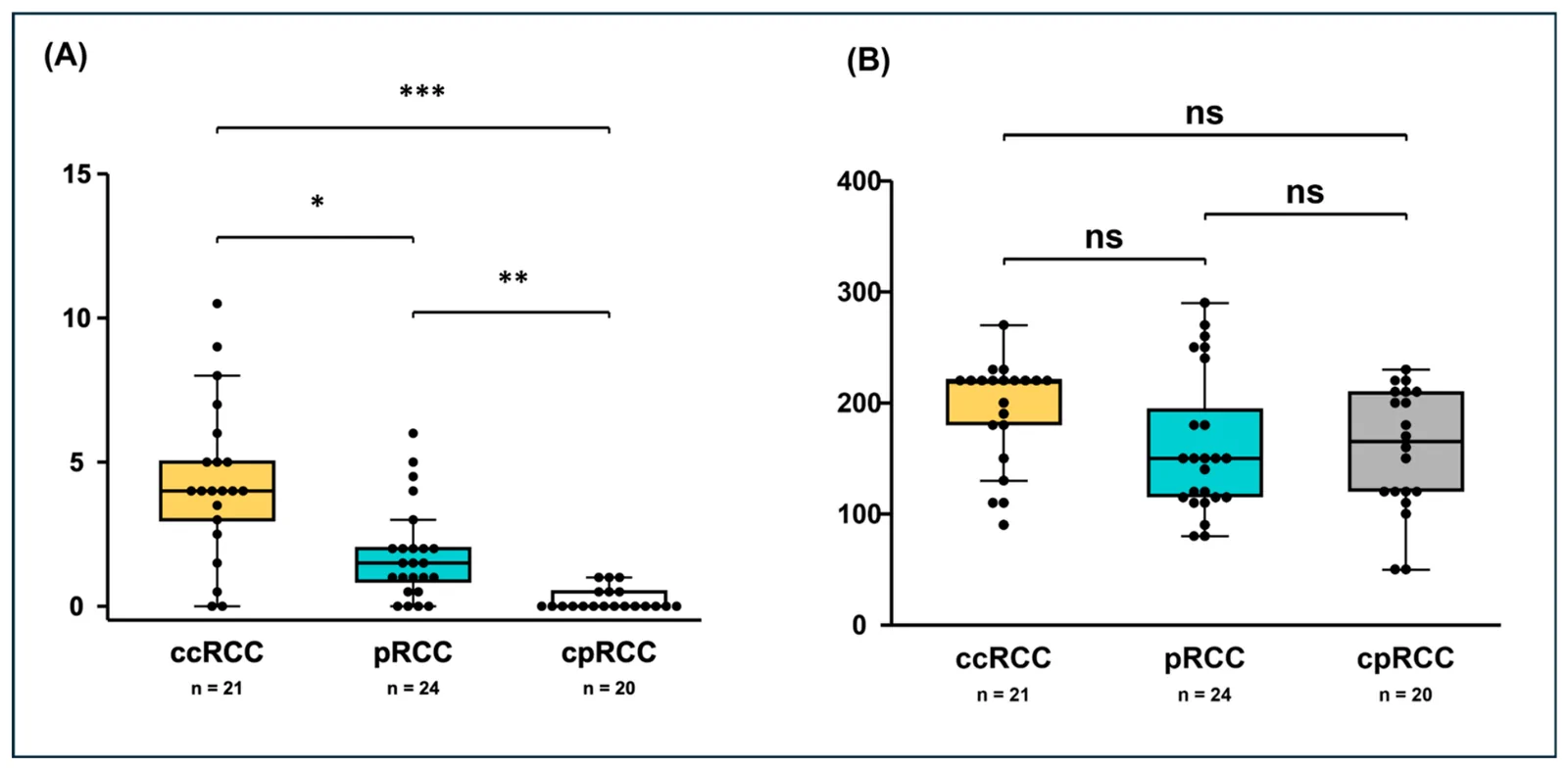
FAP and c-MET expression differ across renal cell carcinoma (RCC) subtypes. (**A**) FAP expression was significantly higher in clear cell RCC (ccRCC) than in papillary RCC (pRCC, *p* = 0.040) and chromophobe RCC (cpRCC, *p* = 5.53 × 10^−8^). In addition, pRCC showed a significantly higher FAP expression than cpRCC (*p* = 0.002). Statistical comparisons were done with the Kruskal–Wallis test followed by the Dunn’s post hoc test. (**B**) Global Kruskal–Wallis test showed no significant (ns) differences in cMET expression between the three investigated RCC subtypes (*p* = 0.087). Boxplots show medians and inter quartile ranges (IQRs) with whiskers extending to the most extreme observations within 1.5 × IQR. Dots indicate individual tumor samples. * *p* < 0.05, ** *p* < 0.01, *** *p* < 0.001.

ccRCC showed significantly higher total IRS scores compared to pRCC (4 [IQR 3.0–5.0] vs. 1.5 [IQR 0.9–2.0], *p* = 0.040) and cpRCC (4 [IQR 3.0–5.0] vs. 0 [IQR 0–0.5], *p* = 5.53 × 10^−8^). In addition, FAP expression was significantly higher in pRCC than in cpRCC (IRS 1.5 [IQR 0.9–2.0] vs. 0 [IQR 0–0.5], *p* = 0.002). This ranking is also evident from the difference in the proportion of RCC subtypes without FAP expression (IRS = 0; ccRCC: 9.5%, pRCC: 16.7%, cpRCC: 70%). Subtype-dependent IRS values of FAP staining are shown in Figure 4A.

### 3.4. c-MET Expression in Different Subtypes of RCC

In all cases of RCC, immunoreactivity for c-MET was detected both on the membrane and in the cytoplasm of tumor cells. No tumor sample was entirely negative for c-MET, but staining patterns varied notably. The median H-score in ccRCC was 220 (IQR 180–220), which was not significantly different from expression in cpRCC (median H-Score 165, IQR 120–210; *p* = 0.169) or pRCC (median H-score 150, IQR 115–195; *p* = 0.159). There was also no significant difference between the H-scores of cpRCC and pRCC (*p* = 1.000). Subtype-dependent H-scores of c-MET staining are shown in Figure 4B.

## 4. Discussion

In this study, we performed a comparative immunohistochemical analysis of FAP and c-MET expression across the three major histological subtypes of RCC (ccRCC, pRCC, cpRCC) to determine their potential value as theranostic targets. Our preliminary results suggest distinct and potentially complementary expression patterns of FAP and c-MET across RCC subtypes, supporting further evaluation of their potential theranostic relevance in RCC.

In line with previous findings, FAP expression was strictly confined to the tumor microenvironment with predominant localization to RCC-associated fibroblasts and tumor-associated vasculature while absent in normal renal parenchyma [46]. The observed high tumor-specific expression of FAP makes it a promising target for molecular imaging and radionuclide therapy in a theranostic approach [25].

Our preliminary data indicate an RCC subtype-dependent FAP expression pattern with the highest FAP levels observed in ccRCC, intermediate expression in pRCC and minimal expression in cpRCC. Interestingly, a recent study reported a corresponding gradient in RCC subtype-associated prognosis with 5-year overall survival rates of 62.7% for ccRCC, 74.3% for pRCC and 82.2% for cpRCC [47]. These observations are in line with previous studies, which characterized ccRCC as a FAP-rich tumor and found a significant association between high FAP expression and decreased survival [48,49]. Consistent with these findings, the proportion of advanced T3-stage disease in our cohort was more than 12-fold higher in RCCs with high FAP expression (39.4%) than in those with low FAP expression (3.1%). Conversely, T1-stage tumors were substantially more frequent in the RCC group with low FAP expression (81.3% vs. 51.5%). However, due to cohort-specific immunoreactivity thresholds and small subgroup sizes, formal statistical analysis of this association was not possible and should be conducted in larger cohorts.

While the exact biological implications of these observations remain unclear, our findings suggest that differences in FAP expression across RCC subtypes reflect underlying differences in FAP-mediated tumor-stroma interactions. As FAP is involved in stromal activation, extracellular matrix remodeling and suppression of effective antitumoral immune responses, it seems likely that high FAP expression contributes to differences in tumor–host interactions, aggressive tumor behaviour, and impaired prognosis [17,50]. In agreement, we observed pronounced FAP expression at the tumor invasion front, particularly in ccRCC. Nevertheless, the biology of FAP-expressing cells is incompletely understood and the mechanisms underlying differential FAP expression among RCC subtypes require further investigation [51].

From a theranostic perspective, our findings are highly relevant as immunohistochemically detected FAP expression was shown to correlate with FAPI PET/CT uptake across multiple cancer types [52]. However, these data were derived from a mixed tumor cohort and did not specifically address RCC subtypes.

It was previously suggested that overall FAP expression in RCC may be lower and more heterogeneous than in several other tumor entities, potentially resulting in variable tracer uptake, moderate detection rates on imaging and reduced therapy effects of FAP-based approaches in RCC [21,26,27,53]. Our results help contextualize these findings by indicating that FAP expression is not uniformly distributed across histological RCC subtypes. The high prevalence and intensity of FAP expression observed in ccRCC suggest that this subtype could be of particular interest for further evaluation of FAP-targeted imaging and therapeutic approaches. Conversely, the lower expression observed in cpRCC might limit the applicability of such strategies in this specific subtype. These preliminary findings therefore offer a basis for considering histological RCC subtypes in the future development and clinical evaluation of FAP-targeted theranostics. This is underscored by recent findings from a small pioneer study in which FAP radiotracer uptake was higher in ccRCC than in pRCC (cpRCC was not represented in that cohort) [26].

c-MET is a receptor tyrosine kinase encoded by the MET gene that binds hepatocyte growth factor and regulates cell growth and survival. In RCC, dysregulation of the MET pathway has been implicated in tumor growth, progression and metastasis [31]. Accordingly, c-MET inhibitors such as cabozantinib are widely used in RCC therapy, and c-MET represents an interesting target for theranostic approaches [32,33,34].

In our study, c-MET expression was detected in all RCC samples, with both membranous and cytoplasmic staining in tumor cells across all RCC subtypes. Interestingly, we found no statistically significant difference in extent and intensity of c-MET expression between the three investigated RCC subtypes. This may seem counterintuitive given the known role of MET alterations in tumorigenesis, especially in a subset of pRCCs [31,54]. However, it is important to remember that protein detection by immunohistochemistry does not necessarily correspond to functional MET pathway activation or the presence of MET gene alterations. Notably, a recent preclinical study found no correlation between MET gene alterations and c-MET protein expression [55]. Therefore, while the broad presence of c-MET protein across RCC subtypes observed in our study supports the feasibility of targeting c-MET protein for molecular imaging, the widespread c-MET expression should not be interpreted as evidence of therapeutically relevant MET pathway alterations.

Consistent with its physiological role in tissue regeneration and homeostasis, c-MET was also expressed in normal renal structures, particularly in tubular epithelium and vascular components [29]. This physiological background expression may limit the use of c-MET as an imaging target in RCC due to low tumor-to-background contrast. In addition, potential side effects through off-target tissue uptake may hamper the therapeutic use of c-MET-targeted radioligands. To date, only a few c-MET-targeted probes (GE-137, ^18^F-AH113804, ^68^Ga-EMP-100 and [^68^Ga]Ga-NOTA-PEG_2_-Glc-MetP) have been investigated in small clinical trials [34,56,57,58]. Although these early imaging studies did not report tracer-related adverse events, they involved only small numbers of participants and were conducted at relatively low diagnostic rather than therapeutic doses. Given the physiological c-MET expression in healthy renal parenchyma and substantial renal uptake reported for ^18^F-AH113804 and ^68^Ga-EMP-100, future studies evaluating c-MET-targeted radioligand therapies must carefully monitor renal radiation exposure and potential nephrotoxicity, especially with repeated administration of high radioligand doses.

The main limitation of our study is the relatively small overall cohort size and the small number of cases within the individual RCC subgroups, which limits the robustness of the observed associations and the reliability of statistical analyses. The retrospective single-center design represents an additional limitation and may further affect the generalizability of our findings. Furthermore, although IRS and H-score are established semi-quantitative scoring systems for immunohistochemical biomarker assessment and are widely used in research and diagnostic pathology [40,59], the absence of interobserver validation limits scoring reproducibility and cohort-specific thresholds are not easy to compare across studies. Prospective multicenter studies are needed to validate our results, assess interobserver variability, and establish clinically relevant cut-offs for FAP and c-MET immunoreactivity.

Since our study lacked paired molecular imaging or autoradiography data, the proposed implications for FAP- and c-MET-based theranostics are extrapolated solely from immunohistochemical findings. Although recent studies have reported associations between immunohistochemically detected FAP and c-MET expression and corresponding molecular imaging findings [52,55], such correlations have not yet been systematically established across RCC subtypes. While our results provide a foundation for further comparative research, studies integrating RCC-subtype-specific tissue-based analyses with paired molecular imaging are needed to validate our findings in vivo and to determine the clinical relevance of FAP and c-MET as potential theranostic targets.

## 5. Conclusions

Our preliminary findings suggest that FAP and c-MET exhibit distinct expression patterns across RCC subtypes. While FAP showed a subtype-dependent expression profile and was localized to the tumor microenvironment, c-MET was broadly expressed in both tumor cells and normal renal tissue. These findings provide a rationale for further investigation of FAP- and c-MET-based subtype-specific theranostic strategies in RCC. In particular, the observed FAP expression patterns warrant further evaluation of FAP as a potential imaging and therapeutic target in ccRCC and pRCC, whereas the broader distribution of c-MET expression supports its investigation as a potential theranostic target across multiple RCC subtypes. Prospective multicenter studies with larger cohorts combining standardized pathology assessment with molecular imaging and clinical outcome data will be required to determine the clinical and theranostic relevance of our findings.

## Figures and Tables

**Table 1:** Clinical and pathological characteristics of FAP stained tumor samples.

	ccRCC (*n* = 21)		pRCC (*n* = 24)		cpRCC (*n* = 20)	
	FAP Low	FAP High	FAP Low	FAP High	FAP Low	FAP High
	*n* = 7	*n* = 14	*n* = 11	*n* = 13	*n* = 14	*n* = 6
Age at diagnosis (years)						
Median (Range)	75 (55–80)	71 (37–82)	77 (54–82)	68 (47–83)	64 (54–90)	66 (36–77)
Sex						
Male (%)	4 (57)	10 (71)	10 (91)	13 (100)	8 (57)	5 (83)
Female (%)	3 (43)	4 (29)	1 (9)	0 (0)	6 (43)	1 (17)
T stage						
T1 (%)	6 (86)	10 (71)	9 (82)	5 (38)	11 (79)	2 (33)
T2 (%)	1 (14)	0 (0)	1 (9)	1 (8)	3 (21)	2 (33)
T3 (%)	0 (0)	4 (29)	1 (9)	7 (54)	0 (0)	2 (33)
T4 (%)	0 (0)	0 (0)	0 (0)	0 (0)	0 (0)	0 (0)
Lymph node status						
pNx (%)	7 (100)	14 (100)	10 (91)	11 (85)	13 (93)	6 (100)
pN0 (%)	0 (0)	0 (0)	0 (0)	1 (8)	1 (7)	0 (0)
pN1 (%)	0 (0)	0 (0)	1 (9)	1 (8)	0 (0)	0 (0)
Lymphovascular invasion						
L0 (%)	7 (100)	14 (100)	10 (91)	12 (92)	14 (100)	6 (100)
L1 (%)	0 (0)	0 (0)	1 (9)	1 (8)	0 (0)	0 (0)
Vascular invasion						
V0 (%)	7 (100)	12 (86)	10 (91)	12 (92)	14 (100)	5 (83)
V1 (%)	0 (0)	2 (14)	1 (9)	1 (8)	0 (0)	1 (17)
Perineural invasion						
Pn0 (%)	7 (100)	14 (100)	11 (100)	13 (100)	14 (100)	6 (100)
Pn1 (%)	0 (0)	0 (0)	0 (0)	0 (0)	0 (0)	0 (0)
Distant metastasis						
M0 (%)	7 (100)	14 (100)	11 (100)	12 (92)	14 (100)	6 (100)
M1 (%)	0 (0)	0 (0)	0 (0)	1 (8)	0 (0)	0 (0)
Residual status						
Rx (%)	0 (0)	2 (14)	0 (0)	0 (0)	0 (0)	0 (0)
R0 (%)	7 (100)	12 (86)	11 (100)	12 (92)	13 (93)	6 (100)
R1 (%)	0 (0)	0 (0)	0 (0)	1 (8)	1 (7)	0 (0)
WHO/ISUP Grading						
G1 (%)	2 (29)	3 (21)	2 (18)	4 (31)	NA	NA
G2 (%)	4 (57)	7 (50)	8 (73)	3 (23)	NA	NA
G3 (%)	1 (14)	4 (29)	1 (9)	5 (38)	NA	NA
G4 (%)	0 (0)	0 (0)	0 (0)	1 (8)	NA	NA

ccRCC, clear cell renal cell carcinoma; pRCC, papillary renal cell carcinoma; cpRCC, chromophobe renal cell carcinoma; WHO/ISUP Grading, WHO Classification of Tumors/International Society of Urological Pathology histological grading of renal cell tumor types according to [37]; NA, not applicable (as histological grading is not recommended for cpRCC) [36]. Stratified comparisons are presented descriptively; given the limited subgroup sizes, no formal statistical testing was performed.

**Table 2:** Clinical and pathological characteristics of c-Met stained tumor samples.

	ccRCC (*n* = 21)		pRCC (*n* = 24)		cpRCC (*n* = 20)	
	c-MET Low	c-MET High	c-MET Low	c-MET High	c-MET Low	c-MET High
	*n* = 9	*n* = 12	*n* = 11	*n* = 13	*n* = 10	*n* = 10
Age at diagnosis (years)						
Median (Range)	66 (37–80)	75 (55–82)	68 (48–78)	61 (47–83)	60 (36–76)	69 (54–90)
Sex						
Male (%)	6 (67)	8 (67)	10 (91)	13 (100)	8 (80)	5 (50)
Female (%)	3 (33)	4 (33)	1 (9)	0 (0)	2 (20)	5 (50)
T stage						
T1 (%)	9 (100)	7 (58)	8 (73)	6 (46)	6 (60)	7 (70)
T2 (%)	0 (0)	1 (8)	0 (0)	2 (15)	4 (40)	1 (10)
T3 (%)	0 (0)	4 (33)	3 (27)	5 (38)	0 (0)	2 (20)
T4 (%)	0 (0)	0 (0)	0 (0)	0 (0)	0 (0)	0 (0)
Lymph node status						
pNx (%)	9 (100)	12 (100)	9 (82)	12 (92)	9 (90)	10 (100)
pN0 (%)	0 (0)	0 (0)	0 (0)	1 (8)	1 (10)	0 (0)
pN1 (%)	0 (0)	0 (0)	2 (18)	0 (0)	0 (0)	0 (0)
Lymphovascular invasion						
L0 (%)	9 (100)	12 (100)	9 (82)	13 (100)	10 (100)	10 (100)
L1 (%)	0 (0)	0 (0)	2 (18)	0 (0)	0 (0)	0 (0)
Vascular invasion						
V0 (%)	9 (100)	10 (83)	9 (82)	13 (100)	9 (90)	10 (100)
V1 (%)	0 (0)	2 (17)	2 (18)	0 (0)	1 (10)	0 (0)
Perineural invasion						
Pn0 (%)	9 (100)	12 (100)	11 (100)	13 (100)	10 (100)	10 (100)
Pn1 (%)	0 (0)	0 (0)	0 (0)	0 (0)	0 (0)	0 (0)
Distant metastasis						
M0 (%)	9 (100)	12 (100)	10 (91)	13 (100)	10 (100)	10 (100)
M1 (%)	0 (0)	0 (0)	1 (9)	0 (0)	0 (0)	0 (0)
Residual status						
Rx (%)	1 (11)	1 (8)	0 (0)	0 (0)	0 (0)	0 (0)
R0 (%)	8 (89)	11 (92)	10 (91)	13 (100)	10 (100)	9 (90)
R1 (%)	0 (0)	0 (0)	1 (9)	0 (0)	0 (0)	1 (10)
WHO/ISUP Grading						
G1 (%)	3 (33)	2 (17)	1 (9)	5 (38)	NA	NA
G2 (%)	6 (67)	5 (42)	7 (64)	4 (31)	NA	NA
G3 (%)	0 (0)	5 (42)	3 (27)	3 (23)	NA	NA
G4 (%)	0 (0)	0 (0)	0 (0)	1 (8)	NA	NA

ccRCC, clear cell renal cell carcinoma; pRCC, papillary renal cell carcinoma; cpRCC, chromophobe renal cell carcinoma; WHO/ISUP Grading, WHO Classification of Tumors/International Society of Urological Pathology histological grading of renal cell tumor types according to [37]; NA, not applicable (as histological grading is not recommended for cpRCC) [36]. Stratified comparisons are presented descriptively; given the limited subgroup sizes, no formal statistical testing was performed.

## Data Availability

The original contributions presented in this study are included in the article. Further inquiries can be directed to the corresponding author.
